# Targeted Programming of the Lymph Node Environment Causes Evolution of Local and Systemic Immunity

**DOI:** 10.1007/s12195-016-0455-6

**Published:** 2016-06-27

**Authors:** James I. Andorko, Joshua M. Gammon, Lisa H. Tostanoski, Qin Zeng, Christopher M. Jewell

**Affiliations:** 1 Fischell Department of Bioengineering, University of Maryland, 8228 Paint Branch Drive, College Park, MD 20742 USA; 2 Department of Microbiology and Immunology, University of Maryland School of Medicine, 685 West Baltimore Street, Baltimore, MD 21201 USA; 3 Marlene and Stewart Greenebaum Cancer Center, 22 S. Greene St., Baltimore, MD 21201 USA

**Keywords:** Lymph node, Vaccine, Adjuvant, Microparticle and nanoparticle, Cancer, Immunotherapy

## Abstract

Biomaterial vaccines offer cargo protection, targeting, and co-delivery of signals to immune organs such as lymph nodes (LNs), tissues that coordinate adaptive immunity. Understanding how individual vaccine components impact immune response has been difficult owing to the systemic nature of delivery. Direct intra-lymph node (*i.LN.*) injection offers a unique opportunity to dissect how the doses, kinetics, and combinations of signals reaching LNs influence the LN environment. Here, *i.LN.* injection was used as a tool to study the local and systemic responses to vaccines comprised of soluble antigen and degradable polymer particles encapsulating toll-like receptor agonists as adjuvants. Microparticle vaccines increased antigen presenting cells and lymphocytes in LNs, enhancing activation of these cells. Enumeration of antigen-specific CD8^+^ T cells in blood revealed expansion over 7 days, followed by a contraction period over 1 month as memory developed. Extending this strategy to conserved mouse and human tumor antigens resulted in tumor antigen-specific primary and recall responses by CD8^+^ T cells. During challenge with an aggressive metastatic melanoma model, *i.LN*. delivery of depots slowed tumor growth more than a potent human vaccine adjuvant, demonstrating local treatment of a target immunological site can promote responses that are potent, systemic, and antigen-specific.

## Introduction

Historically vaccine design has focused on generating potent, specific immune responses. However, equally important for vaccines aimed at persistent and emerging diseases, is the need to better control the nature of the immune responses that are generated. For example, in the context of cancer vaccination, tumor-specific CD8^+^ T cells that exhibit memory-like characteristics and proliferate at very high rates might help overcome the immunosuppressive tumor microenvironment.^10^,^33^ Even vaccines aimed at well controlled pathogens—such as flu—could benefit from formulations that offer better immunomodulatory capabilities, in this example, by conferring increased production of mucosal antibodies.^7^ Another area of intense research along these lines is in the exploitation of new adjuvants—such as toll like receptor agonists (TLRas) that stimulate pathogen-detecting inflammatory pathways. These molecules can be delivered alone, or in combination to create polarizing or synergistic effects.^6^,^26^,^50^,^51^,^55^ Better understanding of the effects of specific vaccine components, adjuvants, and carriers, along with knowledge of how these agents work together, would help support the design of more effective vaccines.

Lymph nodes (LNs) are tissues that initiate, maintain, and regulate adaptive immune response, and are thus critical targets for vaccines and immunotherapies. At these sites, antigen presenting cells (APCs) display antigens to T and B cells with the same specificity to mount antigen-specific effector function.^14^ Thus the local signals integrated in LNs help define the specificity, magnitude, and nature of the resulting systemic responses. A key hurdle facing new vaccines and immunotherapies is efficiently targeting these sites.^30^ For example, to effectively prime lymphocytes against a specific antigen, both the antigen and an adjuvant or other stimulatory immune signal need to be localized to the same tissue, while the combinations and relative concentrations of vaccine components dramatically impact the characteristics of this response. Unsurprisingly, significant interested has developed in strategies that allow more efficient delivery to LNs and more precise control over the local environment in these tissues.

To address the challenges above, many reports in the past several decades have investigated biomaterial carriers (e.g., polymer particles,^25^,^49^ liposomes 15,21,32,48) that encapsulate or adsorb combinations of antigens and adjuvants.^2^ The tunable sizes, particulate nature, and ability to co-deliver cargos make these vehicles attractive as vaccine formulations that can be injected and drain to LNs or can be carried there by APCs.^18^ Particle size plays a major role in the efficiency and route by which these vaccines reach LNs,^42^ an area that has been heavily investigated.^2^,^18^ While many exciting approaches have been reported, even those that generate robust immune responses are limited in the control they provide over the routes or doses by which particles reach LNs after injection. Instead, vaccines generally rely on passive draining through lymphatic vessels, uptake by APCs and subsequent trafficking to LNs, or more recently, active targeting using receptor/ligand interactions.^2^,^18^ Thus, a relatively small faction of the total injected dose actually reaches LNs,^19^,^42^ increasing the required dose in some cases, or preventing efficacious response in others. These effects are also important since some vaccine or immunotherapy components have toxic or inflammatory effects that limit the dose or frequency of administration.

A consideration specific to biomaterial carriers is the growing list of polymers, such as poly(lactic-co-glycolic acid) (PLGA), polystyrene, and others,^2^,^3^,^37^,^47^ that exhibit intrinsic inflammatory effects even in the absence of other immune signals.^2^ PLGA, for example, is used in countless vaccine and immunotherapy studies, but can activate the inflammasome and increases stimulatory response to TLRas.^47^ While these are characteristics that can be harnessed, they can also complicate vaccine research because of the increased complexity resulting from “carrier-effects” that alters how the immune system responds to antigens or other vaccine components. A better understanding of how immune signals—and their biomaterial carriers—interact with the local LN microenvironment, and how these interactions direct systemic immunity would help improve vaccine performance, while also contributing to more rational vaccine design strategies.

We recently developed a strategy to deposit biomaterial vaccine depots directly in LNs of mice using intra-lymph node (*i.LN.*) injection.^3^,^4^,^22^ This platform allows direct control over delivery of vaccine components to LNs, and sustained release of encapsulated cargo within these tissues. In our previous work, we discovered *i.LN.* delivery of microparticles (MPs) encapsulating adjuvant generate more potent responses than nanoparticles or soluble adjuvant because these large particles are better retained in LNs.^22^ Therefore, we sought to use *i.LN.* injection of adjuvant-loaded MPs as a tool to study the evolution of these local and systemic responses over time in mice. We demonstrate that *i.LN.* deposition of vaccine depots consisting of PLGA MPs loaded with a TLR3a and suspended in soluble ovalbumin (OVA) antigen increases the number of APCs and lymphocytes in LNs over the course of 7 days. Treatment does not alter the relative composition of these compartments, but does increase the activation of resident APCs. A single treatment with these vaccine depots expands antigen-specific CD8^+^ T cells locally in treated LNs and systemically in peripheral blood, evolving from a potent effector response at day 7 to a memory response by day 28. We also show this approach is generalizable: *i.LN.* injection of vaccine depots loaded with either PolyIC or CpG—potent TLRas being explored in human trials—and then mixed with conserved human melanoma antigens potently expand tumor-specific CD8^+^ T cells. These effects correlate with slowed tumor progression during an aggressive challenge with metastatic melanoma. Together this work demonstrates that local programming of distinct LNs with adjuvant depots can be used to drive local alterations that promote immunity that is systemic and antigen-specific.

## Materials and Methods

### Particle Synthesis

Degradable MPs were synthesized *via* a double-emulsion, solvent evaporation technique.^4^,^22^ For lipid stabilized particles, 1,2-dioleoyl-sn-glycero-3-phosphocholine, 1,2-distearoyl-sn-glycero-3-phosphoethanolamine-*N*-[amino(polyethylene glycol)-2000], and 1,2-dioleoyl-3-trimethylammoniumpropane (Avanti Polar Lipids) were prepared at a 60:20:20 mol ratio and dried under nitrogen. 80 mg of PLGA (Sigma) was dissolved with the 5.15 *μ*mol of lipids in 5 mL of dichloromethane. An inner aqueous phase containing 500 *μ*L of water or 5 mg of polyinosinic-polycytidylic acid (PolyIC) (Invivogen) in 500 *μ*L of water was added to this organic phase containing polymer and lipid and sonicated for 30 s at 12 W to form the first emulsion. This emulsion was then added to 40 mL of water, homogenized for 3 min at 16,000 rpm, and then allowed to evaporate overnight while stirring to remove any excess organic solvent. Particles stabilized with poly(vinyl alcohol) (PVA, Sigma) were formed as above by removing lipids and replacing the second water phase with a 2% w/v solution of PVA. For particles containing CpG (sequence: 5′ T-C-C-A-T-G-A-C-G-T-T-C-C-T-G-A-C-G-T-T 3′, IDT), 3 mg of CpG in 500 *μ*L of water was used for the first aqueous phase. After overnight stirring, all particle formulations were passed through a 40 *μ*m cell strainer to remove any large aggregates and collected* via* centrifugation (5000×*g*, 5 min, 4 °C). Supernatants were removed and particles were washed three times with 1 mL of water then suspended in water or PBS for animal studies, or lyophilized and stored at 4 °C prior to use. For preparation of fluorescently-labeled particles, 5 *μ*L of DiI (Invitrogen) was added to the organic phase.

### Particle Characterization

Particle diameter was determined using an LA-950 laser diffraction analyzer (Horiba). Zeta potential was measured using a Malvern Zetasizer Nano ZS90. PolyIC and CpG loading levels were determined* via* UV/Vis spectrophotometry after hydrolyzing a known mass of lyophilized particles overnight in 0.2 M NaOH. Absorbance values were compared to standard curves of known concentrations of PolyIC or CpG to determine a mass of cargo per mass of polymer.

### i.LN. Injection

For each animal study, a small region of fur was removed from the lateral hind quarter of 4–6 week old C57BL6 mice (The Jackson Laboratory) by shaving the area and applying a mild depilatory. Tracer dye (Evans Blue) was then injected subcutaneously (*s.c.*) on each side of the tail base as previously reported.^3^,^4^,^22^ After allowing 16 h for the tracer dye to drain to the inguinal LNs for visualization, a 31G insulin needle was used to inject 10 *μ*L containing the indicated treatment into each inguinal LN. For visualization of particles in LNs, 1 mg of DiI labeled MPs were injected. For model antigen studies, vaccinations consisted of 1 mg of particles encapsulating ~8.5 *μ*g PolyIC/mg MPs suspended in PBS with 25 *μ*g soluble ovalbumin (OVA, Worthington) (‘PolyIC MP/OVA’), an injection of 1 mg of PLGA MPs with no cargo (‘Empty’), or an injection of buffer alone (‘sham’), as indicated. In experiments comparing PolyIC and CpG depots, equivalent doses of adjuvant encapsulated in MPs were administered *i.LN,* after being suspended in PBS with 25 *μ*g of soluble OVA or soluble Trp2 (SVYDFFVWL, Genscript) antigens. After priming, mice were boosted with soluble vaccine treatments *s.c.* at each side of tail base at day 21, with each injection consisting of 25 *μ*g antigen and 25 *μ*g adjuvant. For studies comparing melanoma antigens (Trp2, hgp100), treatments included 1 mg of particles containing ~3.5 *μ*g CpG/mg MPs suspended in PBS with 25 *μ*g of soluble Trp2 (‘CpG MP/Trp2′) or soluble hgp100 (KVPRNQDWL, Genscript; ‘CpG MP/hgp100′) antigens, or strong pre-clinical vaccine consisting of 50 *μ*g of CpG and 50 *μ*g peptide formulated with montanide ISA 51 (Seppic; ‘Montanide/CpG/Trp2′ or ‘Montanide/CpG/hgp100’). After vaccinating *i.LN.* at day 0, subsequent boosts for MP groups were given at days 15 and 36 post prime and were identical to the prime but administered *s.c.* at the tail base. For the montanide groups, all injections were *s.c.*, but the second boost consisted of soluble Trp2 or soluble hgp100 mixed with CpG (see caption). All animal studies were approved by the University of Maryland IACUC and conducted in accordance with local, state, and federal guidelines.

### Tissue Collection, Processing, and Flow Cytometry

At the indicated times after treatment, LNs were collected from mice, placed in PBS, and processed into single cell suspensions by mechanical dissociation through a 40 *μ*m strainer. Cells were split into three portions. One portion of cells was centrifuged (800×*g*, 5 min, 4 °C) and suspended in FACS buffer (1 × PBS with 1% w/v bovine serum albumin, Sigma) containing 1% DAPI (Invitrogen) and Liquid Counting Beads (BD) to quantify cell viability and enumerate total cell numbers using a FACS Canto II (BD), respectively. The other two portions of cells were washed once with 1 mL of FACS buffer then blocked with Fc Block (anti-CD16/CD32, BD) for 10 min at room temperature to inhibit any non-specific binding. After blocking, one portion of cells was stained for innate cell type and activation with indicated antibodies against cell surface markers including CD11c, F4/80, CD40, CD80, CD86, and I-A/I-E (mouse MHCII). Cells were then washed twice, suspended in FACS buffer, and quantified* via* flow cytometry. The final portion of cells was stained for lymphocyte populations and antigen-specific tetramer levels. First, 25 *μ*L of anti-SIINFEKL tetramer was added and incubated for 30 min at room temperature. Then, 25 *μ*L of antibodies against surface markers including B220, CD3, CD4, and CD8 were added and incubated for 20 min at room temperature. Cells were then washed and evaluated, as above. The frequency of each cell population (percent of parent population) and number of counted cells per identical acquisition volume (80 *μ*L) was evaluated. The B220 antibody was purchased from eBiosciences and all other antibodies were purchased from BD.

### MHC Tetramer Staining of Peripheral Blood

Every 7 days, 100 μL of blood was collected from mice treated as above* via* submandibular bleeding. Red blood cells were removed by adding 1 mL of ACK lysis buffer to the blood, incubating for 3 min, collecting cells* via* centrifugation (800×*g*, 5 min, 4 °C), and repeating with 1 mL of fresh ACK lysis buffer. After the second round of ACK lysis buffer, cells were suspended in FACS buffer, blocked with Fc Block, and stained with a tetramer specific for either SIINFEKL (CD8-epitope of OVA), Trp2, or hgp100 for 30 min at room temperature. All tetramers were purchased from MBL International. Following incubation, cells were stained against surface markers CD3, CD8, CD44, and CD62L for 20 min at room temperature. After washing twice with FACS buffer, cells were suspended in FACS buffer containing DAPI and the percentage of antigen-specific cytotoxic T cells (DAPI^−^, CD8^+^, tetramer^+^) was quantified* via* flow cytometry. To determine generation of central memory T cell phenotypes, tetramer^+^ CD8^+^ cells were gated for CD44^high^/CD62L^high^ populations and compared to the percentage of effector memory T cells (CD44^high^/CD62L^low^).

### Tumor Challenge Studies

In some studies, after treating mice with the indicated vaccines, mice were administered 300,000 B16-F10 cells (ATCC) in 100 *μ*L of 1× PBS *s.c.* at the hind flank. Each day following inoculation, body weight was monitored and tumor burden was calculated as a product of two orthogonal diameters. Mice were euthanized according to IACUC-approved humane endpoints when the aggregate tumor burden reached 150 mm^2^.

### Immunohistochemical Analysis

At indicated time points, inguinal LNs were removed and frozen in OCT compound (Tissue-Tek). Using a Microm HM 550 cryostat (Thermo Fisher Scientific Inc.), 6 *μ*m sections of LNs were cut, transferred to slides, and allowed to dry overnight. LN tissue was then fixed for 5 min in ice-cold acetone then washed in 1× PBS. Samples were then blocked for non-specific binding of secondary antibody using 5% goat and 5% donkey serum in 1× PBS for 30 min. After washing in PBS, tissues were stained for cell surface markers including B220 (eBioscience), CD3 (Abcam), and CD11c (BD) for 1 h at room temperature. After washing twice with PBS, fluorescent secondary antibodies (Jackson Immunoresearch) were added for 45 min then washed three more times. After staining, sections were fixed with 4% paraformaldehyde, washed with PBS, quenched with 1% glycerol in PBS, and washed again before mounting in Prolong Diamond Antifade Mountant (LifeSciences) and imaging using an Olympus IX83 fluorescent microscope. Processing of images was conducted versus an antibody iso-type control and levels were adjusted equally for all similar channels.

### Statistical Analysis

Student’s *t* tests were used in comparison of two groups. One-way ANOVA with a Tukey post-test was used to compare three or more groups, or two-way ANOVA for comparisons over time. In all cases, analyses were carried out with Graphpad Prism (version 6.02). Error bars in all panels represent the mean ± SEM and *p* values ≤0.05 were considered significant. Levels of significance were defined as *p < 0.05; **p < 0.01; ***p < 0.001; ****p < 0.0001.

## Results

### PLGA MPs are Dispersed in LNs Following i.LN. Injection

**Figure 1:**
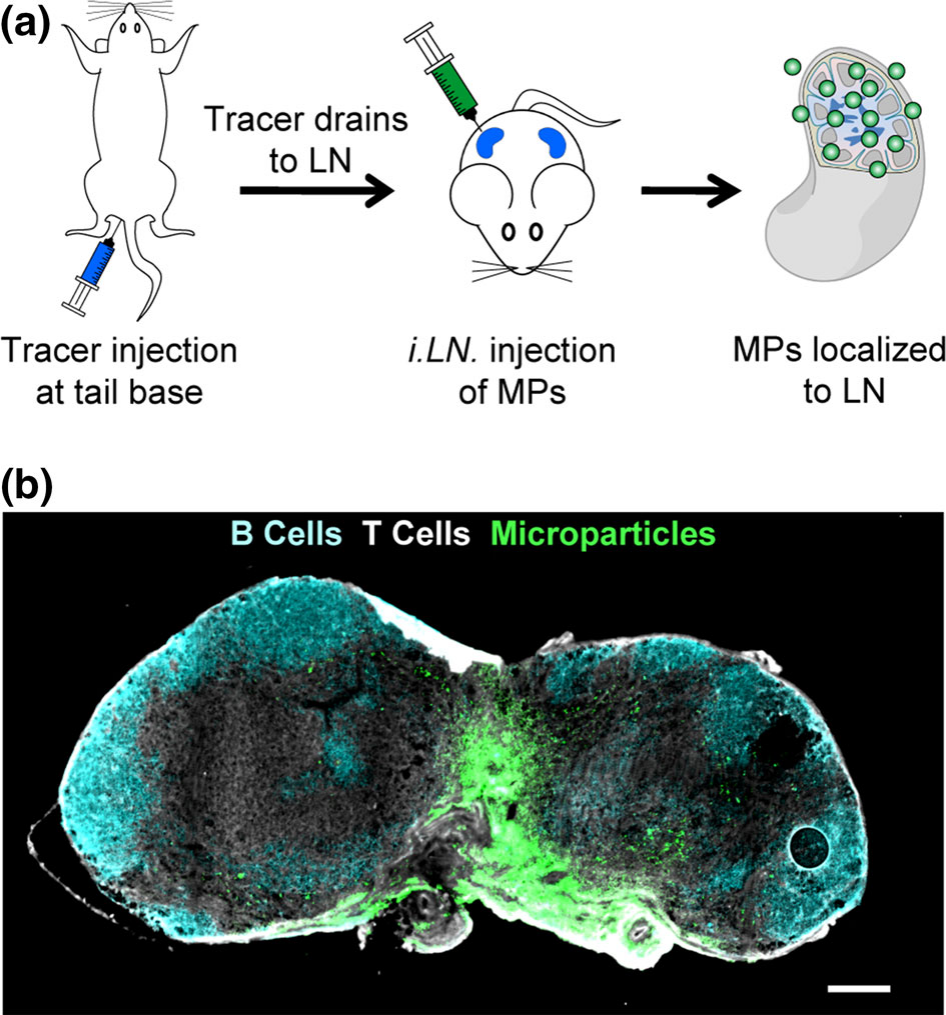
Vaccine depots can be locally deposited in LNs* via* *i.LN.* injection. (a) Schematic depicting *i.LN.* injection of vaccine depots. A tracer dye is injected *s.c.* at the tail base, which then drains to the inguinal LNs allowing visualization of the LN through the skin. Vaccine depots can then be injected into the LN; (b) Histological section of LN 28 days after *i.LN.* injection of fluorescent depots. B cells (B220^+^, cyan), T cells (CD3^+^, white), PLGA MPs (DiI, green). Scale bar = 200 *μ*m.

PLGA MPs were synthesized* via* a double-emulsion/solvent evaporation technique allowing for the inclusion of negatively charged nucleic acid TLRa adjuvants PolyIC or CpG with loading levels of 8.5 *μ*g/mg MP or 3.5 *μ*g/mg MP, respectively (Table 1). Addition of PolyIC led to an increase in particle diameter from 2.2 to 4.3 *μ*m and a shift in zeta potential from 24.9 mV to −23.7 mV; replacement of PolyIC with CpG led to similar shifts (Table 1). To first confirm retention of injected MPs into LNs, we injected DiI-labeled MPs into inguinal LNs of mice using the approach we previously described (Fig. 1a).^3^,^4^,^22^ 28 days after injection, LNs were removed and then stained for B cell (Fig. 1b, cyan) and T cell zones (Fig. 1b, white). Fluorescent microscopy confirmed retention of MPs in the LNs at this time point (Fig. 1b, green).

**Table 1:** Characteristics of adjuvant loaded PLGA-MPs used in *i.LN.* injection studies.

	Diameter (*μ*m)	Loading (*μ*g cargo/mg MP)	Zeta Potential (mV)
Empty	2.19 ± 0.14	n/a	24.93 ± 0.91
PolyIC	4.26 ± 0.09	8.53 ± 0.46	−23.70 ± 0.71
CpG	4.02 ± 0.14	3.45 ± 0.37	−23.23 ± 2.54

### *i*.LN. Injection of PolyIC MP/OVA Increases the Number of APCs and Lymphocytes in LNs

**Figure 2:**
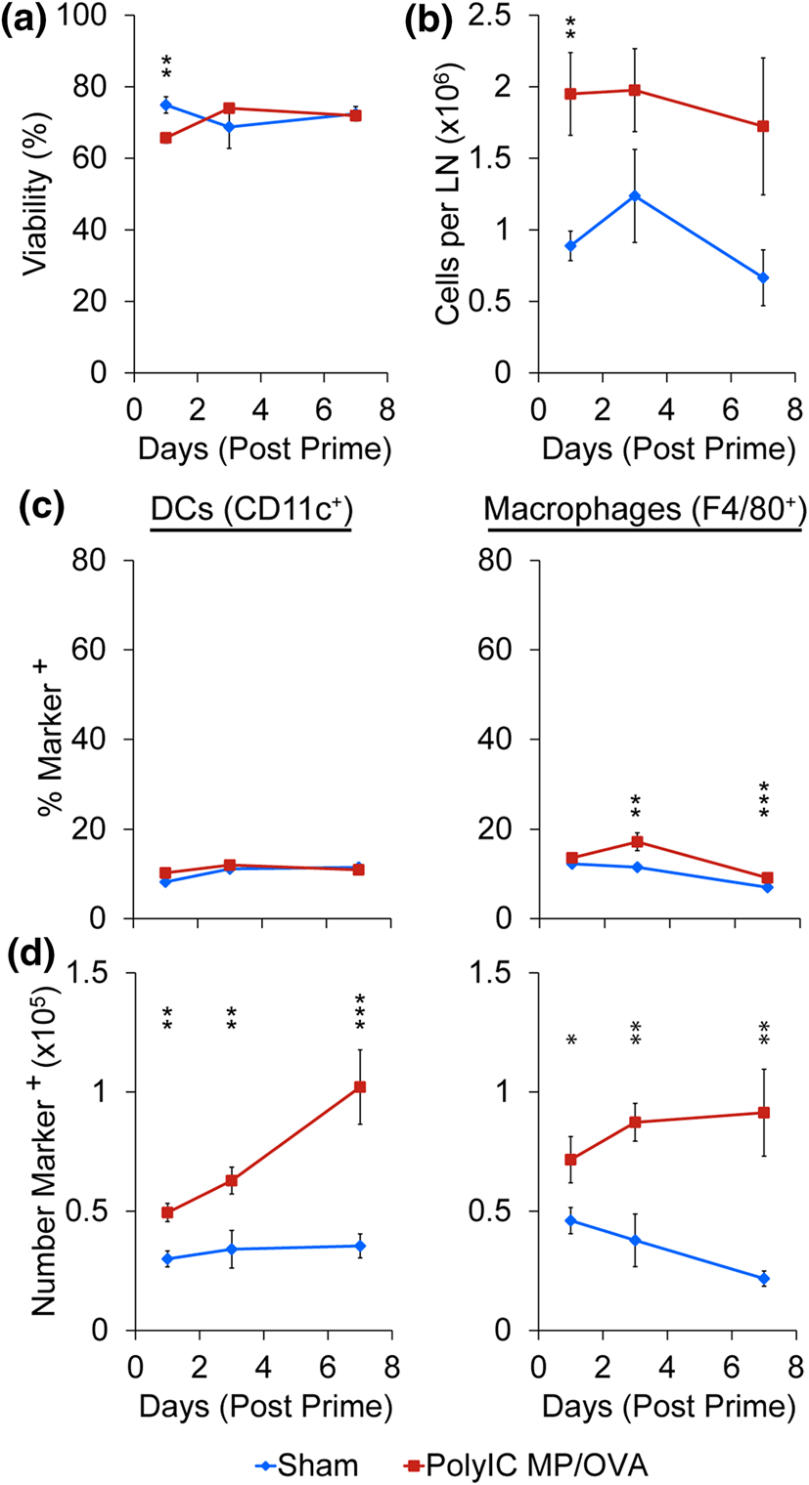
*i.LN.* injection of PolyIC MP/OVA depots increases innate cell numbers in the LNs without affecting cell viability. (a) Viability and (b) total number of LN cells after *i.LN.* injection of PolyIC MP/OVA depots or a sham injection of PBS at days 1, 3, and 7. (c) Percentage of total LN cells which are DCs (CD11c^+^) and macrophages (F4/80^+^) and (d) number of DCs and macrophages in LNs counted in an identical acquisition volume (80 *µ*L). *n* = 9–10 LNs per group with bars depicting mean ± SEM. (*p < 0.05; **p < 0.01; ***p < 0.001).

**Figure 3:**
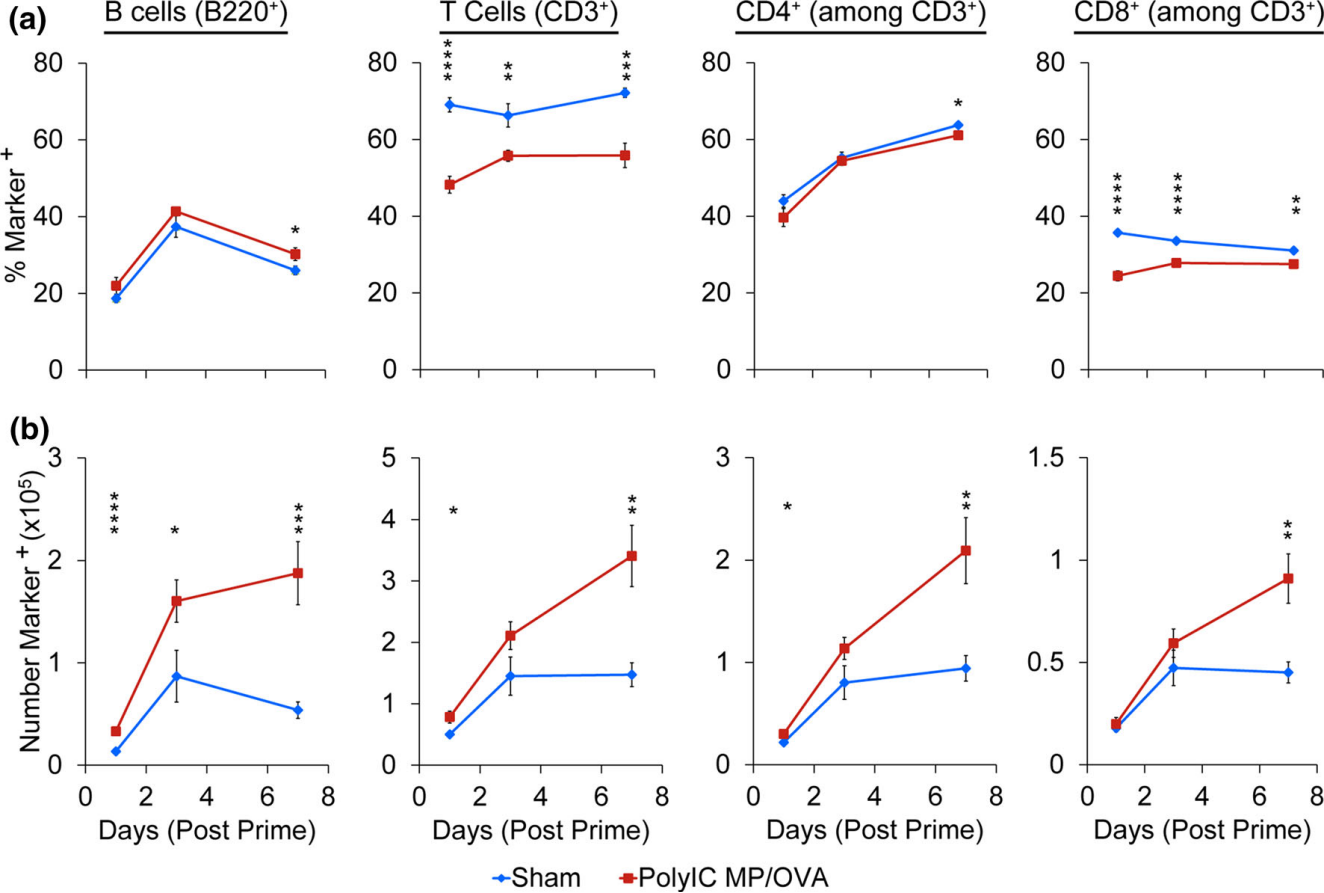
*i.LN.* injection of PolyIC MP/OVA depots increases total number of T and B lymphocytes within LNs. (a) Percentages and (b) total numbers of B cells (B220^+^), T cells (CD3^+^) as well as CD4^+^ T cells (CD3^+^/CD4^+^) and CD8^+^ T cells (CD3^+^/CD8^+^) in LNs after *i.LN.* injection of PolyIC MP/OVA depots or a sham injection of PBS at days 1, 3, and 7. Numbers are counted in an identical acquisition volume (80 *µ*L). *n* = 9–10 LNs per group with bars depicting mean ± SEM. (*p < 0.05; **p < 0.01; ***p < 0.001; ****p < 0.0001).

**Figure 4:**
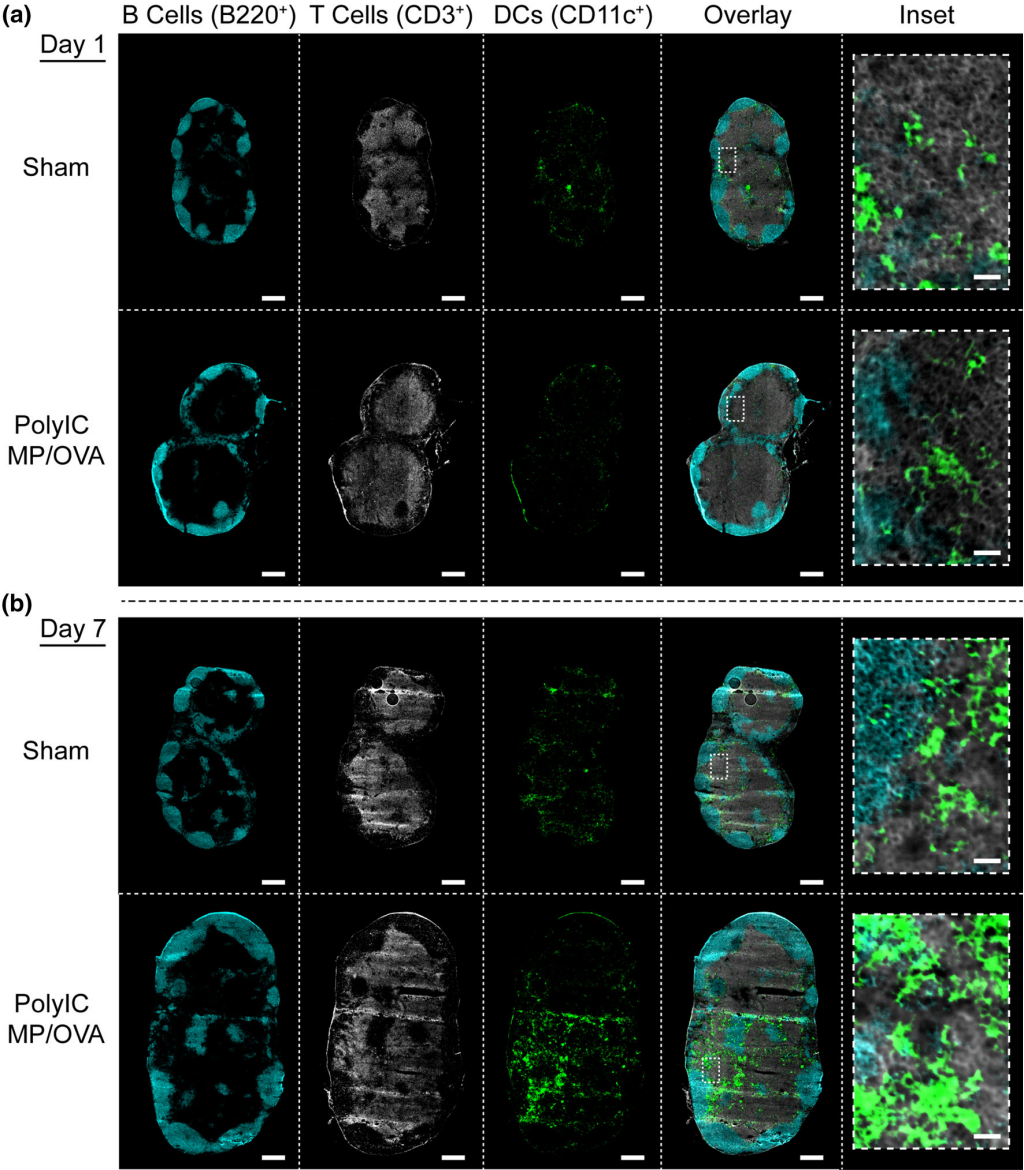
Increased LN size and DC numbers in LNs occurs by day 7 after *i.LN.* injection of PolyIC MP/OVA depots. Histological staining of LNs for B cells (B220^+^, cyan), T cells (CD3^+^, white), and DCs (CD11c^+^, green) in LNs 1 day (a) and 7 days (b) after *i.LN.* injection of PolyIC MP/OVA depots or a sham injection of PBS. Scale bar = 400 *μ*m; 20 *μ*m in inset.

After confirming MPs are retained in LNs of mice over 4 weeks, we used *i.LN.* injection to administer a vaccine of PolyIC MPs mixed with soluble OVA (PolyIC MP/OVA), or to administer a buffer injection (sham). Cell viability and the frequency and number of DCs, macrophages, T cells, and B cells in the treated nodes were then monitored over 1 week using identically-treated sets of groups. Following treatment, PolyIC MP/OVA, while slightly diminishing initial cell viability relative to sham, did not impact viability after 1 week (Fig. 2a). Particles did cause an increase in the overall number of cells (Fig. 2b), as well as the volume of each LN (discussed below), with nodes treated with PolyIC MP/OVA exhibiting significantly more cells per LN than the sham at day 1 (*p* < 0.01); a similar trend was observed over 1 week. In investigating how PolyIC MP/OVA treatment influenced innate immune cell populations, we discovered the frequency of DCs (CD11c^+^) did not significantly change over 1 week, while a slight elevation in macrophage (F4/80^+^) frequency was observed (Fig. 2c). However, the number of each of these cell types (normalized to equivalent tissue cell suspensions) increased over time, with significantly more DCs (*p* < 0.001) and macrophages (*p* < 0.01) accumulating in the LNs over 7 days following PolyIC MP/OVA injection (Fig. 2d). Similarly, we observed modest changes in the frequency of lymphocytes in the B cell (B220^+^) and T cell (CD3^+^; CD3^+^/CD4^+^; CD3^+^/CD8^+^) compartments relative to sham injections (Fig. 3a). However, enumeration of the number of lymphocytes again revealed PolyIC MP/OVA increased the number of cells in each population, with the maximum difference between groups occurring 7 days after the immunization. Immunohistochemical staining of the LNs at 1 day (Fig. 4a) and 7 days (Fig. 4b) after injection confirmed the increased total number of cells, indicated by the increased area evident in each section; all sections are presented at the same scale. These studies also qualitatively confirmed the increased DC levels we measured in response to PolyIC MP/OVA treatment relative to sham, and the increase in DC number as a function of time. These trends are illustrated in the insets of Fig. 4b at day 7 (i.e., sham vs. PolyIC MP/OVA) and the insets of Figs. 4a and 4b for PolyIC MP/OVA (i.e., day 1 vs. day 7), respectively.

### PolyIC MP/OVA Treatment Activates LN-Resident APCs

**Figure 5:**
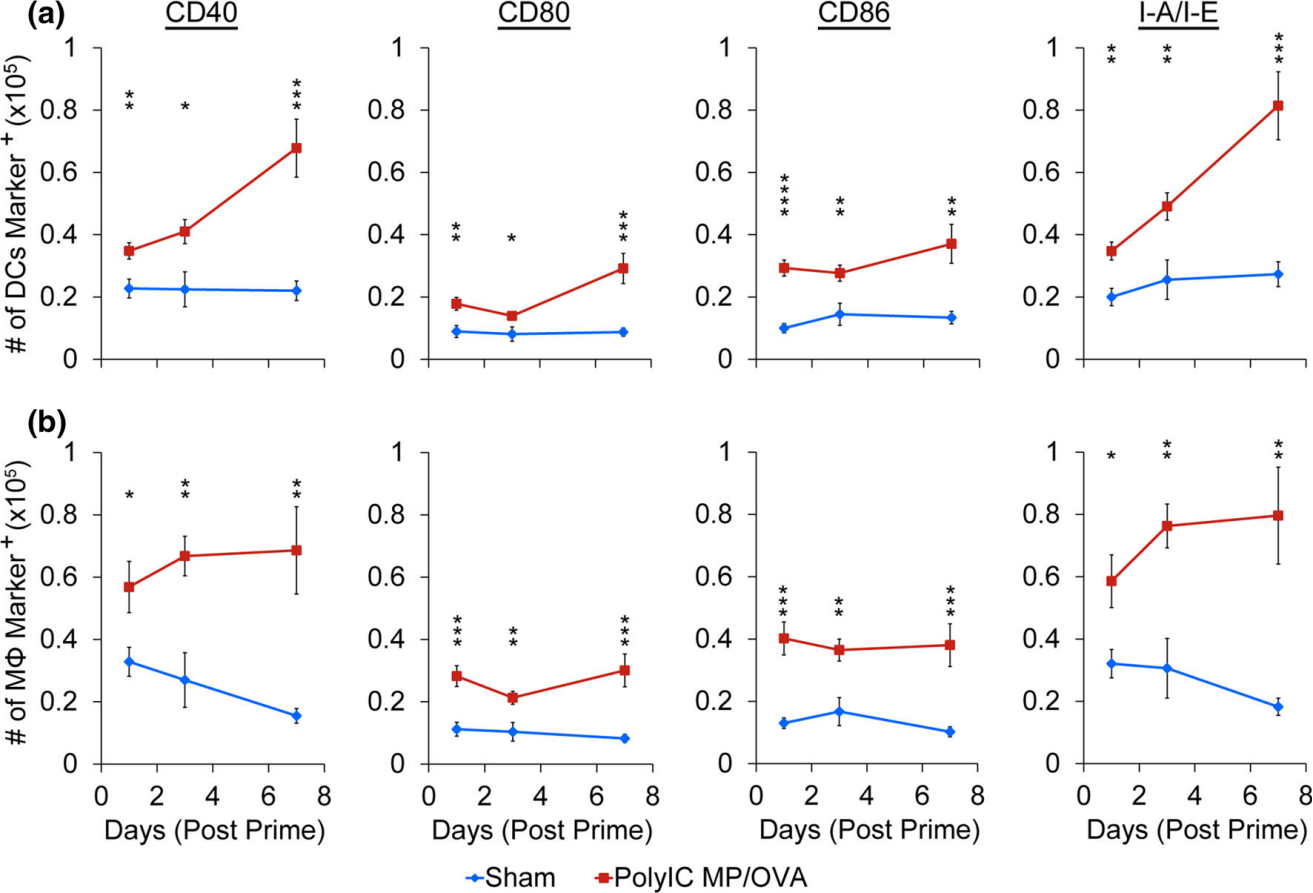
PolyIC MP/OVA depots injected *i.LN.* drive prolonged increase in surface activation marker expression in DCs and macrophages. Number of DCs (a) and macrophages (b) in LNs expressing activation markers CD40, CD80 CD86 and I-A/I-E at 1, 3, and 7 days after *i.LN.* injection of depots. Numbers are counted in an identical acquisition volume (80 *µ*L). *n* = 9–10 LNs per group with *bars* depicting mean ± SEM. (*p < 0.05; **p < 0.01; ***p < 0.001; ****p < 0.0001).

After determining that *i.LN.* treatment with PolyIC MP/OVA increases the number of APCs, we tested if these populations exhibited an increased activation state by staining for surface activation markers associated with co-stimulation and antigen presentation (i.e., CD40, CD80, CD86, I–A/I–E). In all cases, PolyIC MP/OVA caused a significant increase in the number of cells positive for each marker compared to the sham injected control (Fig. 5a). Interestingly, the number of activated DCs increased over time with the highest levels of each marker occurring 7 days after treatment (Fig. 5a, red). The macrophage population exhibited similar activation effects (Fig. 5b). However, compared to DCs, which showed increases in the number of cells expressing each marker over time, only CD40 and I-A/I-E increased as a function of time. Macrophage expression levels of CD80 and CD86—while higher than levels in sham-injected nodes—remained at a near-constant, elevated level over 1 week.

### Local Changes in APC Function Drive Local and Systemic Antigen-Specific CD8^+^ T Cell Response

**Figure 6:**
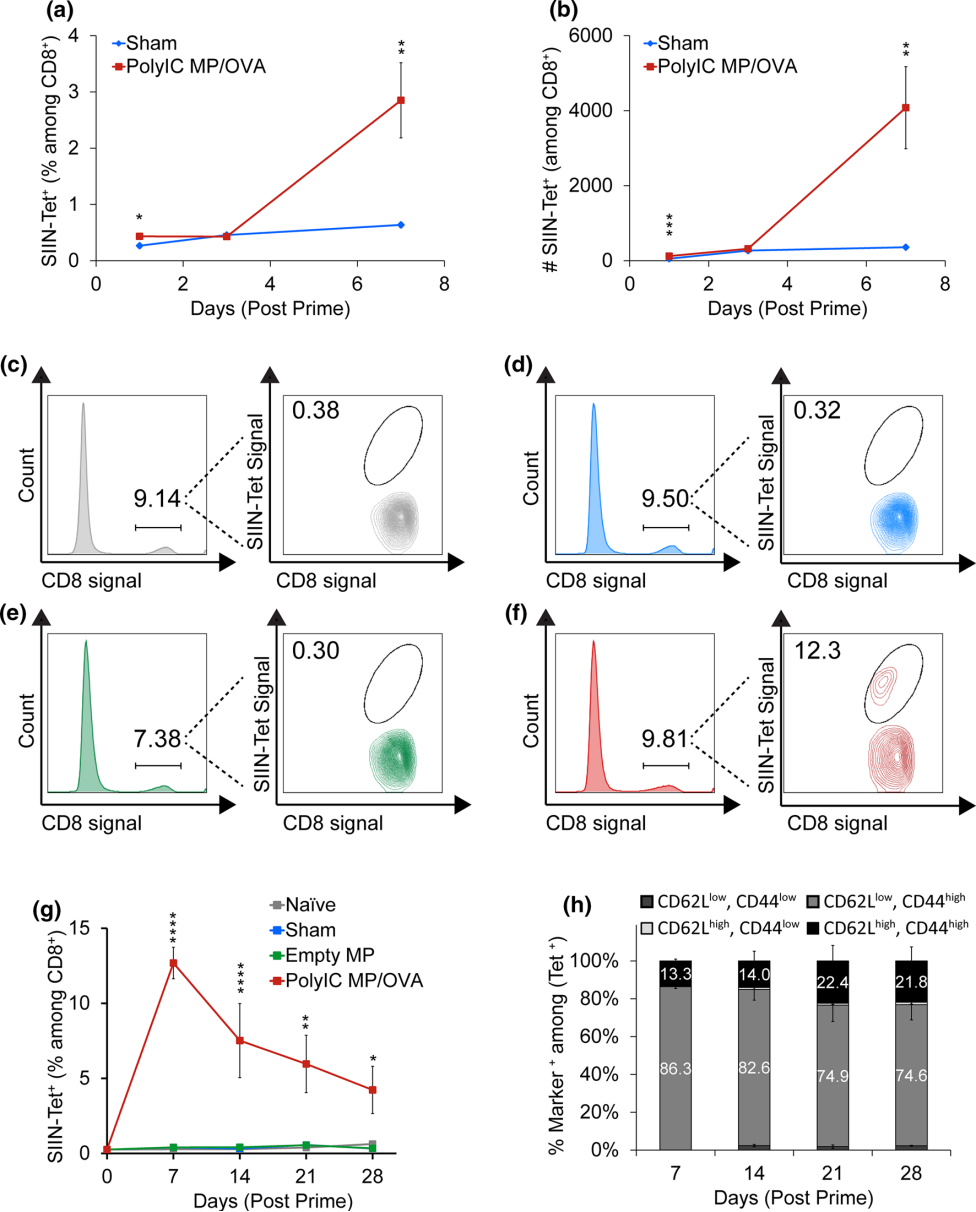
*i.LN.* injection of depots drives antigen-specific T cell responses locally in LNs and systemically in the periphery. (a) Percentage and (b) numbers of SIINFEKL-tetramer^+^ CD8^+^ T cells in LNs at 1, 3 and 7 days after *i.LN* injection of PolyIC MP/OVA depots or a PBS sham injection. Numbers are counted in an identical acquisition volume (80 *µ*L). *n* = 9–10 LNs per group with *bars* depicting mean ± SEM. (**p < 0.01; ***p < 0.001) Mice were immunized *i.LN.* with PolyIC MP/OVA depots, Empty MPs, a sham injection of PBS or left untreated (naïve), and leukocytes from peripheral blood were stained for SIINFEKL-tetramer^+^ CD8^+^ T cells weekly starting 7 days after immunization. Representative flow cytometry plots illustrating the gating scheme for SIINFEKL tetramer staining of untreated mice (c), mice immunized *i.LN.* with a sham injection of PBS (d), Empty MPs (e), or PolyIC MP/OVA depots (f) 7 days after treatment. (g) Mean percentage of SIINFEKL-tetramer positive T cells and (h) percentage of SIINFEKL positive T cells with effector (CD62L^low^/CD44^high^) or memory phenotypes (CD62L^high^/CD44^high^) in mice from treatment groups detailed in (c–f). *n* = 8 mice for Day 0, *n* = 10 mice per group at Day 7, and *n* = 4–5 mice per group for Days 14–28. (*p < 0.05; **p < 0.01; ***p < 0.001; ****p < 0.0001).

We next used MHC-I tetramer staining to investigate if the local activation we observed drove generation of antigen-specific T cells, both in treated nodes and systemically. Analysis of LNs after treatment revealed that vaccinating with PolyIC MP/OVA increased both the frequency and number of antigen-specific CD8^+^ T cells within the LN (Figs. 6a and 6b). While the sham injection (Figs. 6a and 6b, blue) remained at a constant, low level, the PolyIC MP/OVA treated mice exhibited a significant (*p* < 0.01) increase in SIINFEKL-specific T cells 7 days after priming. To investigate how these local changes to the LN microenvironment impacted systemic changes in antigen-specific responses, mice were treated with either PolyIC MP/OVA, empty MPs, a sham injection, or left untreated. After vaccination on Day 0, blood was collected weekly and SIINFEKL tetramer staining was used to determine the percentage of antigen-specific CD8^+^ T cells circulating in peripheral blood. Figures 6c–6f depicts representative flow cytometry plots showing the gating scheme applied to samples from naïve (Fig. 6c, gray), sham (Fig. 6d, blue), empty MP (Fig. 6e, green), or PolyIC MP/OVA (Fig. 6f, red) treated mice 7 days after immunization. The average SIINFEKL tetramer levels revealed that treatment with PolyIC MP/OVA significantly increased *(p* < 0.0001) systemic levels of SIINFEKL-specific CD8^+^ T cells 7 days after treatment, followed by a prototypical contraction period through day 28 (Fig. 6g). The elevated level of SIINFEKL-specific CD8^+^ T cells at day 28 suggested development of immune memory, which we assessed using common markers for effector T cells and memory T cells among CD8^+^/Tetramer^+^ cells. These studies revealed a nearly twofold increase in the percentage of central memory T cells (CD62L^high^/CD44^high^ among SIINFEKL-specific CD8^+^) and a subsequent decrease in effector memory phenotypes (CD62L^low^/CD44^high^) over this same time (Fig. 6h).

**Figure 7:**
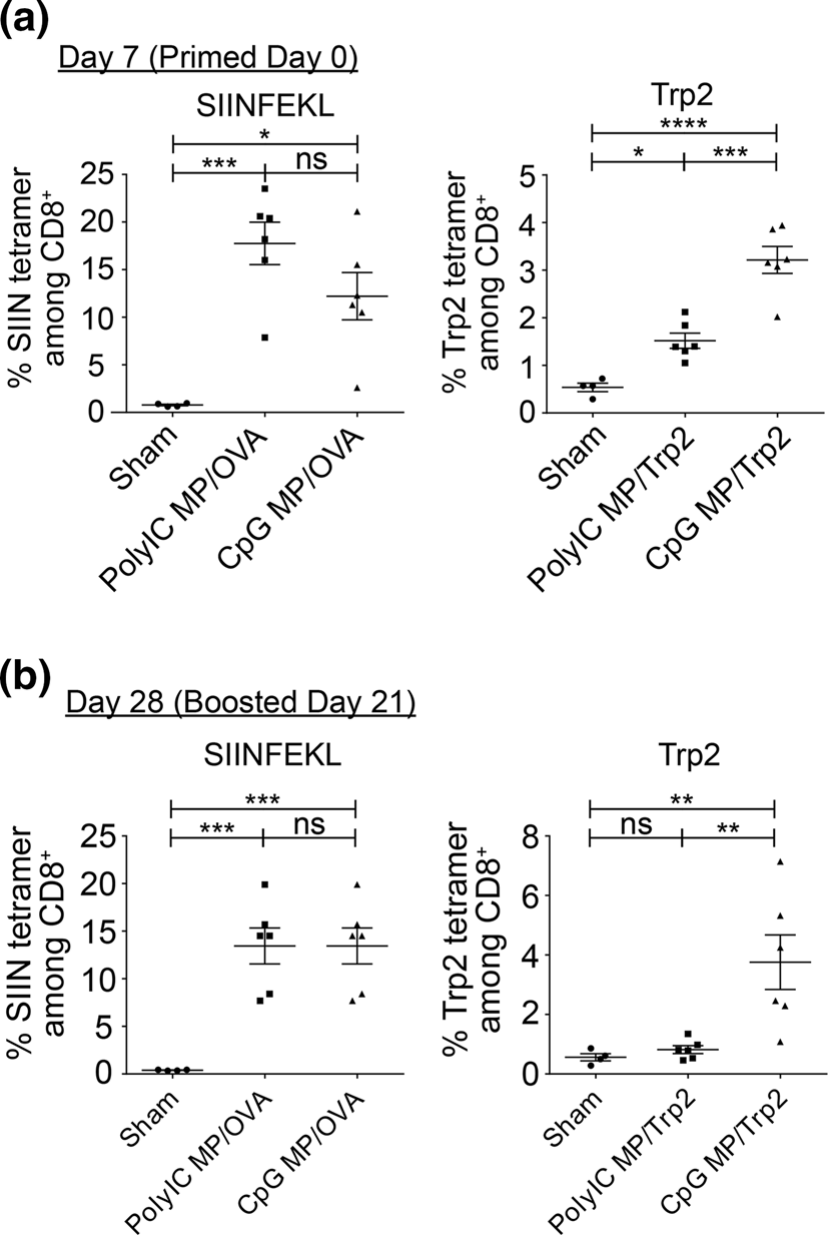
CpG MPs induce superior tumor-specific CTL responses compared to PolyIC MPs. Mice were primed at day 0 *i.LN.* will either PolyIC MPs or CpG MPs, and either a model antigen (OVA) or a melanoma associated antigen (Trp2) in a soluble form. Mice were boosted at day 21, and antigen-specific MHC-I tetramer was used to measure antigen specific CD8^+^ T cell responses compared to a sham injection. (a) 7 days after priming, PolyIC and CpG MPs both induced potent levels of SIINFEKL-specific CD8^+^, but no differences were observed as a function of TLRa. In the Trp2 model, both PolyIC and CpG MPs increased the levels of Trp2-specific CD8^+^ T-cells, with CpG exhibiting a statistically significant increase compared to both the sham and PolyIC MP injections. (b) At day 28, 7 days after the boost, a similar response was seen with a robust response in the OVA model for both PolyIC and CpG MPs, but without dependence on the specific TLRa included in the particles. In the Trp2 studies, only CpG MPs induced a significant, potent recall response. (*p < 0.05; **p < 0.01; ***p < 0.001; ****p < 0.0001).

To test the robustness and modularity of this platform, we next tested if *i.LN.* injection expands antigen-specific T cells with vaccines containing different TLRas or other antigens, in particular, Trp2 peptide—a clinically-relevant tumor associated antigen conserved in murine and human melanoma.^38^ Depots were formulated with either PolyIC or CpG—a potent adjuvant being studied to induce anti-tumor immunity 12,49—and mixed with soluble OVA or Trp2. Mice were immunized *i.LN* at day 0 with vaccine depots encapsulating identical doses of adjuvant, and then boosted at day 21 with soluble vaccine components *s.c.* at the tail base. At days 7 and 28 (7 days after the prime and boost injections), peripheral blood was drawn and MHC-I tetramer staining was used to quantify the percentage of antigen specific CD8^+^ T cells (Trp2 tetramer for Trp2 immunized mice, SIINFEKL tetramer for OVA immunized mice). For mice immunized with OVA vaccine depots both treatments induced very potent antigen-specific responses, but no significant differences were measured between responses induced by CpG MPs and PolyIC MPs at either day (Fig. 7, left). However, in mice treated with Trp2 vaccine depots, a significantly higher level of Trp2 specific CD8^+^ T cells was observed in mice immunized with CpG depots compared to PolyIC depots at both time points (Fig. 7, right).

### Local administration of CpG particles promotes anti-tumor immunity

**Figure 8:**
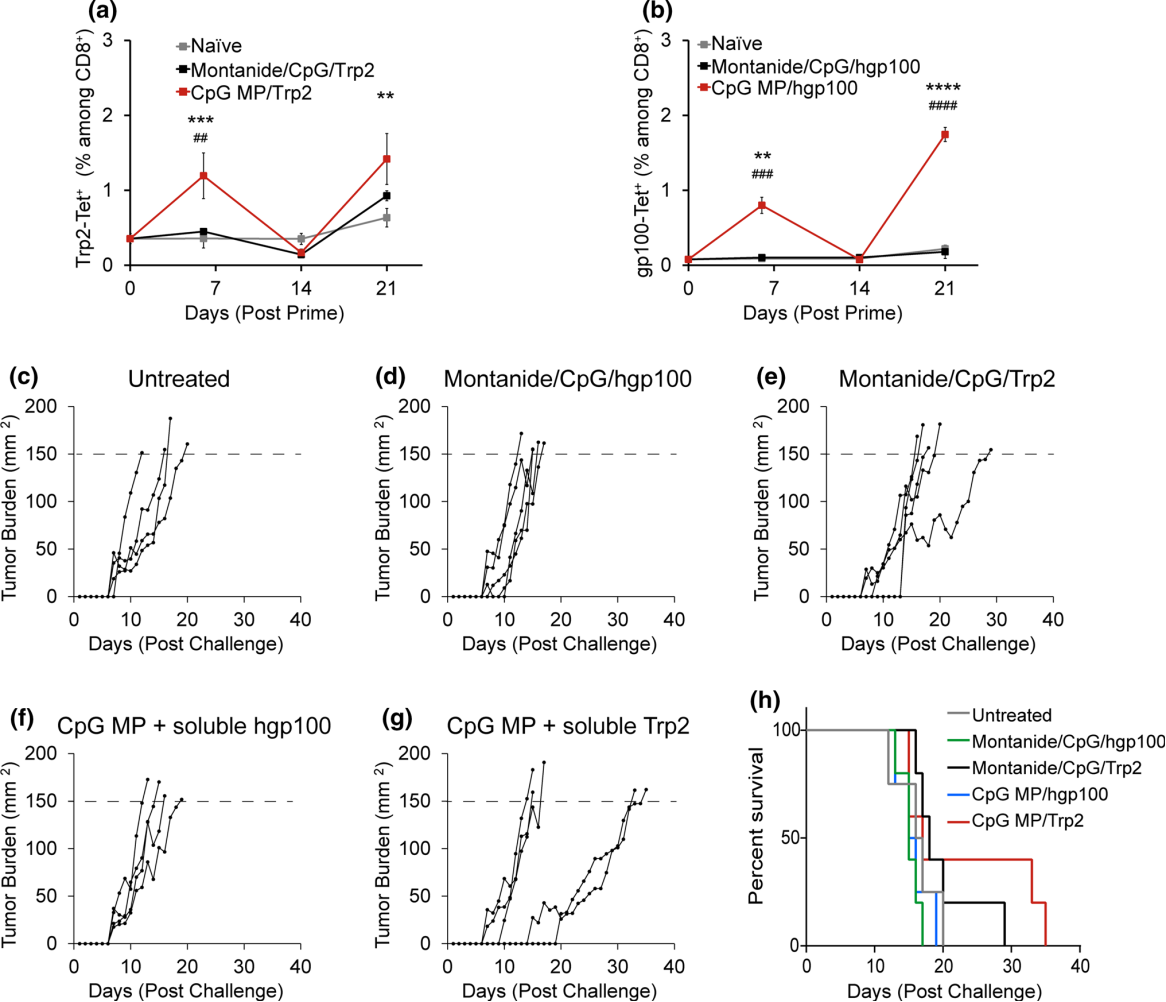
*i.LN.* injection of CpG MP/Trp2 depots promote functional anti-tumor immunity. (a) Mice were left untreated, immunized *s.c.* with Montanide/CpG/Trp2, or immunized *i.LN.* with CpG MP/Trp2, followed by *s.c.* boosts consisting of identical treatments at Day 15. Trp2-tetramer specific T cells were quantified in peripheral blood at 6, 14 and 21 days after immunization. (b) A study conducted using identical treatment regimens as in (a), but including an additional tumor antigen, hgp100. hgp100-specific CD8^+^ T cell responses in peripheral blood were quantified using hgp100 MHC-I tetramer in peripheral blood at 6, 14 and 21 days after immunization. Values indicate mean ± SEM. (**p < 0.01; ***p < 0.001; ****p < 0.0001 between CpG MP groups and naïve; ^##^ *p* < 0.01; ^###^ *p* < 0.001; ^####^ *p* < 0.0001 between CpG MP groups and montanide). (c–h) Mice were left untreated, immunized with Montanide/CpG/hgp100, Montanide/CpG/Trp2, CpG MP/hgp100 (*i.LN.*), or CpG MP/Trp2 (*i.LN.*) followed by *s.c.* boosts at Day 15 and Day 36 as described in the methods. 43 days after the priming injection, mice were challenged with B16-F10 melanoma. Individual tumor traces of untreated mice (c), mice immunized with Montanide/CpG/hgp100 (d), Montanide/CpG/Trp2 (e), CpG MP/hgp100 (f) and CpG MP/Trp2 (g). (h) Percent survival of mice in the groups shown in (c–g).

We next used an aggressive melanoma model—B16-F10—to test the functionality of anti-tumor immunity induced by vaccine depots administered by the *i.LN.* route. Since vaccine depots formulated with CpG promoted superior expansion of Trp2-specific cytotoxic T lymphocytes (CTLs) compared with PolyIC (Fig. 7), we immunized mice with CpG depots containing 3.5 µg of CpG and suspended in either Trp2, or another conserved melanoma antigen, hgp100.^28^,^35^ In these studies, mice were primed on day 0 with either CpG MP/tumor antigen, or as a potent benchmark, 50 *µ*g CpG and tumor antigen emulsified in montanide, one of the strongest adjuvants currently under study.^27^,^54^ Animals were then boosted on day 15 with identical doses and formulations, but all injections were administered *s.c.* as a heterologous prime-boost regimen. MHC-I tetramer staining for either Trp2- or hgp100-specific CD8^+^ T cells revealed formulations containing CpG MPs exhibited significant increases in these populations relative to other groups after both priming and booster injections (Figs. 8a and 8b). After a second boost on day 36, mice were challenged with B16-F10 metastatic melanoma by implantation of 3 × 10^5^ cells *s.c.* at the hind flank. Compared to the untreated group (Figs. 8c and 8h), the mice primed *s.c.* with montanide/CpG/hgp100 (Figs. 8d and 8h) or *i.LN.* with CpG MPs/hgp100 (Figs. 8f and 8h) did not exhibit any therapeutic gains. In contrast, *i.LN* immunization with CpG MP/Trp2 slowed tumor growth, resulting in 40% survival at day 20 (Figs. 8g and 8h), while all untreated mice succumbed by this day (Figs. 8c and 8h). Interestingly, while Montanide/CpG/Trp2 prolonged survival of mice to 29 days after tumor challenge (Figs. 8e and 8h) the effect appeared less potent than those generated by CpG MP/Trp2 vaccine regimens, which survived for up to 35 days. The mean survival was 23.0 ± 4.5 days for the CpG MP/Trp2 treated group, compared to 20.0 ± 2.4 days for the Montanide/CpG/Trp2 treated group, and 16.3 ± 1.7 days for the untreated group, further demonstrating the ability of local LN treatment to promote functional, systemic immunity.

## Discussion

Biomaterials offer a robust platform to co-deliver immune signals, target vaccines to specific tissues, and control delivery kinetics. However, most vaccines have complex formulations with multiple components, and understanding how each component influences the immune response alone or together has been challenging thus far. Previous research has shown that altering material properties can influence and improve the targeting of vaccines to LNs through lymphatic drainage or trafficking within specific APCs after internalization.^20^,^30^,^40^,^41^,^49^*i.LN.* delivery, however, offers a unique opportunity to directly study how the form and combination of signals that ultimately reach LNs impact immune response without the complexities that occur after vaccines are administered by traditional routes. For example, even efficacious vaccines only result in a small fraction of the injected dose reaching the LN and spleen—as little as 0.1%, whereas pre-clinical and clinical trials studying *i.LN.* delivery of soluble vaccines have demonstrated dose-sparing factors as high as 10^6^ relative to common peripheral injection routes.^23^,^45^,^53^ With respect to nanoparticles, past studies have revealed that particles administered along common peripheral routes drain to LNs most efficiently when the diameters are in the range of 20-30 nm, whereas even 100 nm particles drain an order of magnitude less efficiently.^42^ MP drainage relies heavily on APC trafficking.^2^ Our own past findings demonstrate that improved retention of adjuvant in LNs achieved by encapsulation in MPs too large to freely drain from LNs after *i.LN.* injection drives very strong T cell responses compared to equivalent doses of soluble adjuvant administered *i.LN.*, or adjuvant MPs administered peripherally (e.g., in muscle).^22^ In contrast, nanoparticles or soluble adjuvant are retained in LNs at intermediate and low levels, respectively, driving correspondingly lower responses relative to MPs.^22^ Thus, here we used *i.LN.* injection of MPs to add new understanding of how these local treatments alter LN function over time, and how this local evolution impacts systemic immunity.

With respect to local changes in LNs, several of our findings together suggest an adjuvant mechanism underpinned by increased activation of LN-resident APCs. First, we generally observed large difference in the number of immune cells in treated nodes relative to sham injections, with more modest differences in the relative cell compositions. These frequencies—for both innate and adaptive immune cells—were similar to those previously reported in LNs of C57BL6 mice.^34^ Second, we observed persistence of fluorescent MPs for at least 4 weeks (Fig. 1b), and increased activation of LN-resident APCs (e.g., macrophages, DCs) as soon as 1 day after injection. Thus, one important role for the depots appears to be enhanced local APC function that could help increase lymphocyte proliferation and infiltration. The resulting antigen-specific responses showed enhancements consistent with strong T cell response. For example, OVA-specific T cells developed locally in LN over 7 days, by which time a dramatic increase was measured in peripheral blood. This evolution is consistent with primed lymphocytes migrating out of the LNs as they expand against SIINFEKL presented in these sites.^56^ Similarly, a shift towards a central memory phenotype and away from effector response was also observed over time, a goal for effective vaccines.^39^ Interestingly, we did observe that both depots and sham injections caused modest—sometimes, transient—increases in the frequency of B cells and CD4^+^ T cells. Thus, an additional enhancing mechanism could be mild inflammation caused by injection that, for example, could upregulate adhesion molecules (e.g., P-, E-selectin) to better retain circulating T and B cells. The absence of toxicity, and the intact follicular structure of LNs after either sham or adjuvant MP treatment, further supports the compatibility of this strategy for fundamental or applied uses.

The link between the kinetics of vaccine dosing and induction of immune response is well established, with elegant studies demonstrating that increasing dosing regimens drive synergistic immune responses more effectively than equivalent doses administered in a bolus or at evenly spaced equal doses.^24^ This discovery supports the basic premise for delivery of controlled release depots to LNs, as the local dose of vaccine components locally increases in LNs as cargo is released from degrading polymer particles.^22^ Further, while there is significant potential made possible by determining whether vaccine particles loaded with antigen, adjuvant, or both might be most potent for a particular vaccine,^26^ design of adjuvant-loaded particles offer the appeal of “plug-n-play” vaccination whereby the particle is simply mixed with a soluble adjuvant of interest.

We found *i.LN.* injection of adjuvant MPs drove antigen-specific T cell responses against both model antigen (i.e., OVA) and tumor-associated antigens (i.e., Trp2, gp100) mixed with the depots. Interestingly, for OVA, both PolyIC-loaded and CpG-loaded depots performed equivalently, while CpG was more effective in generating responses against tumor-associated antigens. CpG has stimulated great interested in pre-clinical cancer studies owing to effective priming of CTL response.^11^,^12^,^21^,^31^,^49^ Thus, we benchmarked *i.LN.* delivery of CpG MPs mixed with common conserved melanoma antigens, against these same antigens emulsified with CpG and montanide, one of the strongest vaccine formulations under study.^27^,^54^ With respect to both tumor-specific T cell expansion and anti-tumor immunity, *i.LN.* depots were superior to montanide, but interestingly, the dose of CpG in MP formulations (3.5 *µ*g/LN) was 14-fold lower than the 50 *µ*g dose of CpG emulsified in the montanide vaccines. Thus, although the efficacy achieved with *i.LN.* depots in this study was modest (~40% of mice exhibited significantly increased survival), the enhanced performance compared with montanide and this dose-sparing supports the potential of future MP-based vaccines administered to LNs.

There are some considerations that might account for the limited efficacy observed in tumor challenge studies. First, the chosen melanoma model is highly aggressive. Second, general features of the tumor microenvironment likely limit immunogenicity, including suppression and antigen editing that prevents tumor-specific CTLs from maintaining function or recognizing antigens in tumors.^33^,^44^ Third, in our experiments, we observed much higher frequencies of SIINFEKL-specific T cell responses after a single *i.LN.* immunization with OVA depots relative to either melanoma antigen, even after the latter were administered in several booster injections. OVA is a foreign antigen, whereas Trp2 and hgp100 are self-antigens and typically much less immunogenic. Since cross-presentation of minimal epitope peptides such as Trp2 and hgp100—can enhance immunogenicity,^16^,^18^,^29^,^32^ encapsulation of antigen in MPs alone, or in conjunction with adjuvant might offer one route to further improve potency. However, since significant populations of antigen-specific CD8^+^ T cells were generated against either tumor antigen, we speculate more robust responses might improve effectiveness. Along these lines, recent pre-clinical and clinical studies reveal simultaneously activating multiple TLR pathways during cancer therapy can enhance therapeutic efficacy,^1^,^5^,^13^,^52^ suggesting another strategy based on loading of MPs with multiple TLRas.

*i.LN.* delivery of MPs also provides some unique opportunities to impact the tumor microenvironment through appropriate selection of the LN for injection. In our studies we selected the inguinal LN for ease of injection based on our past work, and what has been used in recent human trials involving *i.LN.* delivery of soluble tumor antigens to inguinal LNs.^43^ However, this technique could also be used to target tumor draining lymph nodes (TDLN), sites which have recently been shown to be effective for passive targeting of cancer vaccines.^9^,^10^,^20^,^49^ Remarkably, several landmark studies also demonstrate that both anti-tumor T cells and regulatory T cells (T_REGs_)—cells that suppress anti-tumor response in tumors—are primed in the same LN.^8^,^17^ Thus, direct LN targeting of TDLNs might allow local polarization toward effector cells while also reducing suppressive T_REGs_ that play an important role in maintaining the suppressive tumor microenvironment. This may further provide an opportunity to effectively combat tumors without affecting natural regulatory activity in other distant LNs. It is also possible that targeting TDLNs is not necessary if optimized particles expand tumor-specific cells that are able to migrate to tumors, but further studies will be needed to investigate this possibility. Finally, creating opportunities to overcome the suppressive characteristics of tumors by directly targeting the TDLN, or pairing with exciting new immunotherapies such as checkpoint blockades could also have offer significant potential for cancer vaccination.^36^,^46^

## Conclusion

*i.LN.* injection allows direct control over the dose and combinations of materials administered to LNs, supporting a new approach for studying the impact of vaccines on the LN microenvironment. Here, we demonstrate that a single *i.LN.* injection can lead to dramatic local changes in these tissues, increasing the number and function of both APCs and lymphocytes. The local changes result in systemic, but antigen-specific pro-immune function that provides functional anti-tumor immunity in a melanoma model. Thus, this approach might hold clinical utility for vaccines based on intra-LN controlled release of antigens and adjuvants, while also providing a strategy to evaluate the immunogenicity of biomaterial carriers themselves, or to design carriers loaded with defined combinations of antigens and adjuvants.
